# Biotechnological development of *Trichoderma*-based formulations for biological control

**DOI:** 10.1007/s00253-023-12687-x

**Published:** 2023-07-21

**Authors:** Yolanda Martinez, Javier Ribera, Francis W. M. R. Schwarze, Kevin De France

**Affiliations:** 1 Empa – Swiss Federal Laboratories for Materials Science and Technology, Laboratory for Cellulose and Wood Materials, St. Gallen, Switzerland; 2 Department of Chemical Engineering, Queen’s University, Kingston, Canada

**Keywords:** Biocontrol formulations, Trichoderma, Carrier substances, Microencapsulation, Fungal propagules

## Abstract

**Abstract:**

*Trichoderma* spp. are a genus of well-known fungi that promote healthy growth and modulate different functions in plants, as well as protect against various plant pathogens. The application of *Trichoderma* and its propagules as a biological control method can therefore help to reduce the use of chemical pesticides and fertilizers in agriculture. This review critically discusses and analyzes groundbreaking innovations over the past few decades of biotechnological approaches to prepare active formulations containing *Trichoderma*. The use of various carrier substances is covered, emphasizing their effects on enhancing the shelf life, viability, and efficacy of the final product formulation. Furthermore, the use of processing techniques such as freeze drying, fluidized bed drying, and spray drying are highlighted, enabling the development of stable, light-weight formulations. Finally, promising microencapsulation techniques for maximizing the performance of *Trichoderma* spp. during application processes are discussed, leading to the next-generation of multi-functional biological control formulations.

**Key points:**

• *The development of carrier substances to encapsulate Trichoderma propagules is highlighted.*

• *Advances in biotechnological processes to prepare Trichoderma-containing formulations are critically discussed.*

• *Current challenges and future outlook of Trichoderma-based formulations in the context of biological control are presented.*

## Introduction

Biological control or biocontrol is broadly defined as the use of living organisms (termed antagonists or biological control agents; BCAs) to control pests such as insects, weeds, parasites, pathogenic fungi, or other invasive species. While there are a number of examples of extremely effective and environmentally benign BCAs, the broad biodiversity of pest species necessitates continuous innovation in biological control strategies (Stiling and Cornelissen 2005). In addition, BCAs may have undesired side-effects on non-target organisms within an ecosystem, compelling researchers to thoroughly investigate and understand the interactions of potential BCAs and microbial populations in different soils and crops before widespread commercial use (Massart et al. 2015; Qin et al. 2019). However, despite its many complexities, the concept of biological control has become increasingly important in recent years because of legislative and consumer pressures to phase out or reduce the use of traditional chemical-based pesticides in agricultural practices (Symondson et al. 2002; Fravel 2005; Wang et al. 2015). This has prompted the development of new classes of fungal-based BCAs such as *Trichoderma* spp., *Aspergillus niger*, and *Ampelomyces quisqualis* and bacterial-based BCAs such as *Bacillus* spp., *Agrobacterium radiobacter*, and *Pseudomonas fluorescence*, all of which are highly effective against multiple plant pathogens, do not contaminate the environment, and do not elicit rapid resistance development within target pathogens (Keswani et al. 2016).

**Fig. 1:**
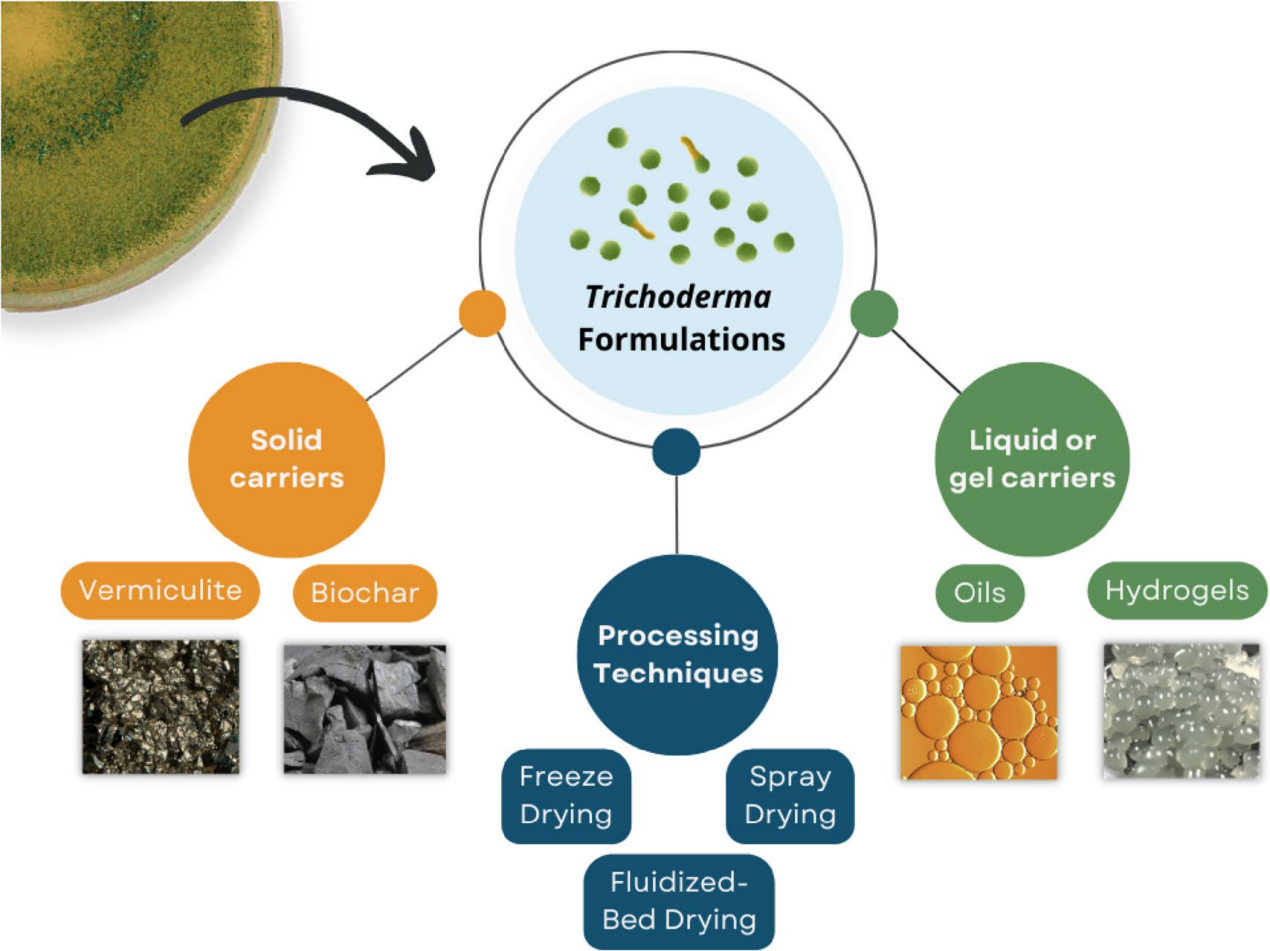
Overview of some common *Trichoderma* carriers and processing techniques highlighted in this review

One of the most widely researched BCAs is *Trichoderma* which includes ubiquitous mesophilic fungi characterized by an outstanding ability to colonize different environments, and as a result, can be found in nearly all soil habitats (Contreras-Cornejo et al. 2016) (Druzhinina et al. 2018). This genus of the family *Hypocreaceae* shows rapid vegetative growth (2 cm day^−1^ or more under ideal conditions) (Kredics et al. 2003, 2004) and produces abundant asexual spores (e.g., *T. viride* has conidia with a diameter of 3–5 µm and chlamydospores with a diameter of 8–10 µm) (Lewis and Papavizas 1984). Conidia are the most common cell type that are produced by asexual reproduction and allow dispersal of the fungus. In general, *Trichoderma* spp. proliferate best by colonizing roots in the plant rhizosphere, and as a result, mycelium will out-compete pathogenic fungi and synergistically enhance plant development (Altomare et al. 1999; Sharma et al. 2012). Several studies have demonstrated the considerable number of mechanisms towards biocontrol used by *Trichoderma* spp. (Manzar et al. 2022; Tyśkiewicz et al. 2022), including mycoparasitism, antibiosis, competition for nutrients/space, tolerance to stress through enhanced root and plant development, solubilization and sequestration of inorganic nutrients, systemic acquired resistance, and enzyme inactivation. As a result, *Trichoderma* spp. have gained considerable commercial interest as BCAs for several common plant diseases including root rot, damping off wilt diseases (Sharma et al. 2009; Elshahawy and El-Mohamedy 2019), and other postharvest diseases, as well as for the preventive treatment of wood against decay fungi (Schubert et al. 2008; Schwarze et al. 2012; Ribera et al. 2017a). Since *Trichoderma harzianum* was first registered by the United States Environmental Protection Agency in 1989 for control of plant diseases (Fravel 2005), *Trichoderma* spp. are the leading commercialized BCA on the global market (Butt et al. 2002). In this review, the use of *Trichoderma* spp. as a prominent BCA is discussed in-depth, with an emphasis on the most recent and groundbreaking innovations in formulation development and biotechnological processing for biocontrol applications (Fig. 1). Proof-of-concept research along with the growing number of commercially available *Trichoderma*-based products demonstrate both the academic and industrial interest in this research field and highlights the current success and positive long-term outlook for *Trichoderma* spp. in biological control.

## Formulations for biocontrol applications

A successful biocontrol formulation containing fungal propagules should be easy to prepare, maintain its functional properties (viability, germination rate, enzymatic activity) during prolonged storage, and facilitate its application on target organisms (Harman and Custis 2006). Faria et al. showed that when *Trichoderma* conidia are immersed in pure water without any carrier substance, the viability of the cells decreases due to a phenomenon called imbibition damage (Faria et al. 2017). This describes the death of hydrated cells due to damage of the cell membrane; to overcome this issue, the use of carrier substances that protect and stabilize the cells must be investigated and included in the formulations of biocontrol products. Besides improving the shelf life of the products, these carrier substances should ideally also enhance the tolerance of the conidia (or other propagules) to environmental conditions such as UV radiation or washout from the application areas. Concentrations of *Trichoderma* conidia in biocontrol products typically range from 1 × 10^5^ to 1 × 10^9^ colony-forming units (CFU) per gram of product (Sokhandani et al. 2016) (Natsiopoulos et al. 2022). Several types of formulations have been developed for the encapsulation and delivery of microbial BCAs (Table 1). Some of these formulations have resulted in commercial *Trichoderma*-based products; a summary of such successful products registered in Europe is highlighted in Table 2. In the European Union, *Trichoderma* products must follow the Regulations (EC) No 1107/2009 in order to be approved, and the list of the current approved products can be consulted in the EU Pesticide database. It is remarkable that most of the current formulations approved for *Trichoderma* spp. products are based on two different formulations: wettable powders, which are dust-forming dry formulations (usually clay and silica) mixed with surfactants that form suspensions when dissolved in water (Kala et al. 2020), and water-dispersible granules, which consist of granular solid particles that disperse or dissolve quickly in water giving a fine particle suspension (G. A. Bell 1998). Typically, *Trichoderma* formulations in the form of wettable powder, granules, emulsions, and suspensions are often used in applications including ground and aerial sprays, root drenching, dipping, seed treatments, irrigation, and hydroponics. On the other hand, formulations in the form of pellets, dry flowables, and other solid-based formulations can be directly applied, such as by incorporating them into the soil during seeding or transplanting (Woo et al. 2014).

**Table 1:** Summary of common formulation types and properties for microbial biopesticide products

Type of formulation	Physical status	Re-suspends in water	Key characteristics	Typical application method
Dusts and powders	Solid	No^*^	Solid carrier material (typically fine mineral particle such as talc, fly ash, clay) ranging from 50 to 100 µm	Direct application to soil/seed dressing
Granules and microgranules	Solid	No^*^	Solid carrier material larger in size (ca. 100–1000 µm)	Direct application to soil
Emulsions	Liquid	Yes	Oil droplets dispersed in water or vice versa (dispersed droplet size ranges from 0.1 to 10 µm)	Spray application/irrigation systems
Concentrated suspensions	Liquid	Yes	Finely ground solid particles (granules or powders) dispersed in water. Particle sizes typically range from 1 to10 µm	Spray application/irrigation systems/seed dressing
Oil dispersions	Liquid	Yes	BCA propagules in solid form dispersed in oil	Spray application/irrigation systems
Encapsulated suspensions	Liquid	Yes	BCA propagules encapsulated in an emulsion or polymer capsule	Spray application/irrigation systems

^*^Some powders and granules can be formulated to effectively disperse/re-suspend in water and are often referred to as wettable powders and water-dispersible granules, respectively. These represent the most successful classes of commercialized formulations

**Table 2:** A summary of current *Trichoderma* biocontrol products commercialized on the European market

Species and strain	Product name	Conidia concentration	Producer	Formulation	Listed shelf life
*T. asperellum* ICC 012 *T. gamsii* ICC 080	Remedier® Tenet®	3 × 10^7^ CFU/g	Isagro S.p.A	Wettable powder	15 months at room temperature
*T. asperellum* T25 *T. atroviride* T11	Tusal®	0.5% w/w of 1 × 10^8^ CFU/g	Certis Europe B.V	Water-dispersible granules	2 years at at 4 °C
*T. asperellum* T34	T34 Biocontrol®	1 × 10^12^ CFU/kg	Biocontrol Technologies SL	Wettable powder	2 years at 4 °C 6 months at 15–20 °C
*T. asperellum* TV1	Xedavir®	2.8% w/w 1 × 10^7^ CFU/g	Xeda Italia S.r.l	Wettable powder	1 year at 4–6 °C 8 months at T < 25 °C
*T. atroviride* I-1237	Esquive® Tri-Soil®	1 × 10^8^ CFU/g 1 × 108 UFC/g	Agrauxine S.A Certis Europe	Wettable powder Wettable powder	1 year at low temperature 4–10 °C 6 months at 20 °C or 18 months at 4 °C
*T. atroviride* IMI 206040 *T. polysporum* IMI 206039	BINAB®T	Min 1 × 10^5^ CFU/g	BINAB Bio-Innovation AB	Wettable powder and granules	2 weeks at 20 °C, 1 year at 4 °C
*T. harzianum* T-22 RIFAI (KRL-AG2)	Trianum-G® Trianum-P®	1% of 1 × 10^8^ CFU/g	Koppert B.V	Water-dispersible granules	6 months at 4–8 °C
*T. atrobrunneum* T720	Avengelus®	1 × 10^7^–1 × 10^9^ CFU/g	MycoSolutions	Water-dispersible granules and suspensions	2 years (granules) at room temperature
*Trichoderma atroviride* strain SC1	Vintec®	1 × 10^10^ UFC/g	Bi-PA	Wettable-dispersible granules	Unspecified

One of the most difficult challenges when developing *Trichoderma* formulations is to identify suitable carrier substances. These substances should allow for high survival rates over time, maintain dormancy/viability of the conidia during prolonged storage, and must not be harmful to the cells themselves. Among the external factors that influence germination of fungal spores, the most important are water availability, temperature, light and pH (Turgeman et al. 2016; Nguyen Van Long et al. 2017). Specifically for *Trichoderma* spp., some studies indicate that conidia require of an exogenous supply of nutrients to facilitate germination and elongation of the hyphae (a phenomena also known as polar growth) (Martín and Nicolás 1970; Šimkovič et al. 2015). In the following sections, the most relevant research on environmental-friendly carriers used to develop *Trichoderma* biocontrol products are summarized, and the current methods for developing light-weight formulations are highlighted.

## Dry formulations

**Fig. 2:**
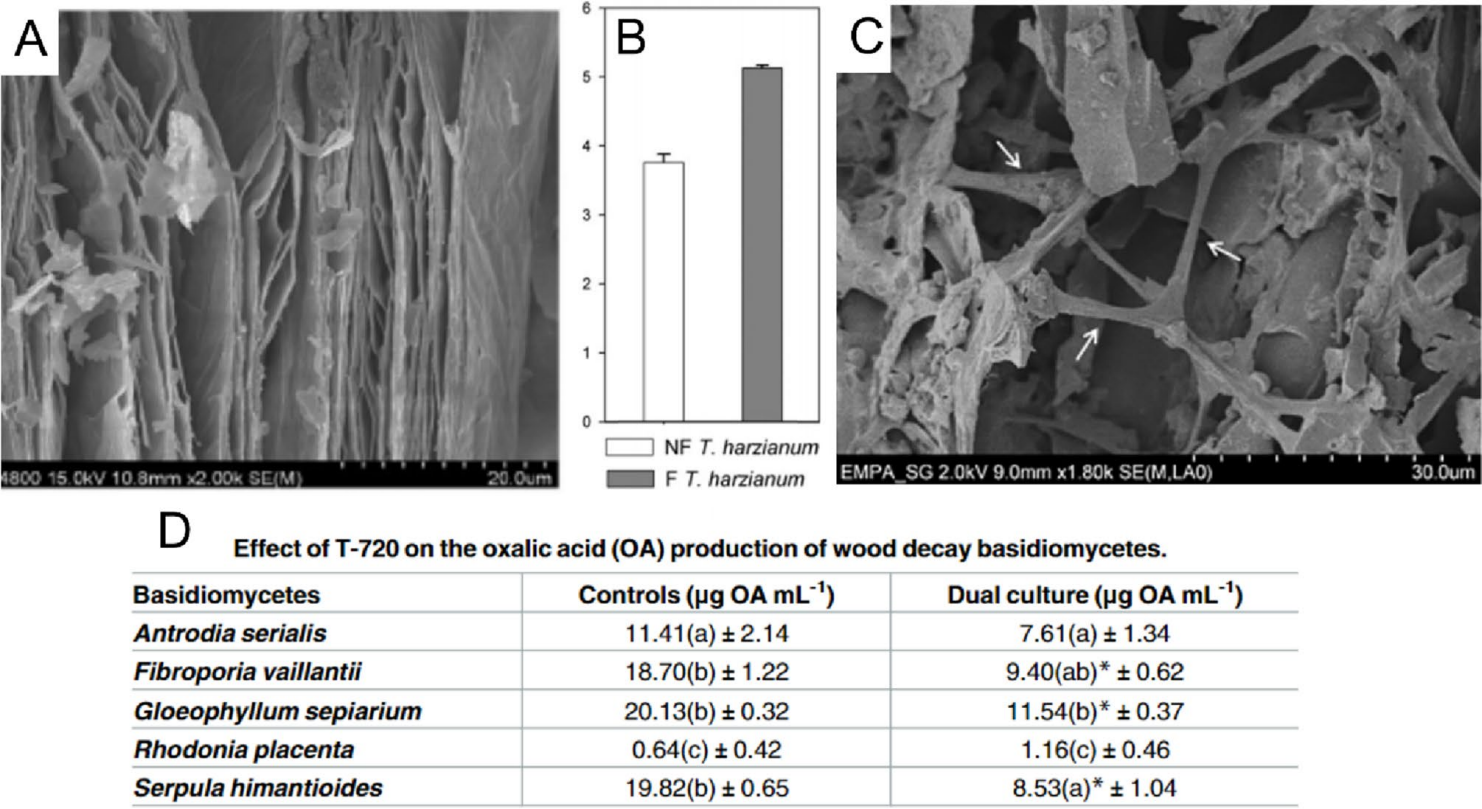
A summary of vermiculite and biochar mineral-based dry formulations. **A** SEM image showing the plate-like structure of solid vermiculite particles; adapted with permission (Deng et al. 2018), copyright Wiley 2018. **B** Viability of *T. harzianum* population after 4 weeks of storage either non-formulated (*NF*) or mixed with a vermiculite-based carrier (*F*); adapted with permission (Martínez-Medina et al. 2009), copyright American Society for Horticultural Science 2009. **C** SEM image showing colonization of biochar by *T. harzianum*, with hyphae indicated by arrows; **D** this formulation demonstrated significant reduction in the production of oxalic acid for several strains of wood decay basidiomycetes; adapted with permission (Ribera et al. 2017b), copyright PLOS 2017

Dry formulations are the most commonly used formulation class in the development of *Trichoderma-*based products, dominating the commercial market. One of the main advantages of dry formulations from a manufacturing perspective is the lower susceptibility to contamination as compared to liquid formulations. Dry formulations are typically mineral-based, which provide a generally high water retention capacity, although grain-based powders have also proved to be useful for mass cultivation and as carrier substances (Elshahawy et al. 2017). Further advantages of mineral-based formulations are the simplicity of the formulation development and the low cost-production of the final products. However, typical drawbacks include a shorter shelf life of the products due to a considerable loss in conidial viability during processing and the generation of dust during packaging and application (Maxim et al. 2014). Critically dust, which contains spores and small mineral particles, can rapidly disperse into the air and potentially lead to respiratory irritation and other health issues (Maxim et al. 2014). Nevertheless, the simplicity, low cost, and overall acceptable efficacy of mineral-based formulations has led to the overwhelming popularity of dry formulations on the commercial market. Some of the most common dry formulations for *Trichoderma* products use carrier substances based on vermiculite or biochar (Fig. 2); these particular formulations are discussed in detail below.

### Vermiculite-based formulations

Vermiculite is a phyllosilicate that belongs to the group of mica minerals. It has a very high water-holding capacity, associated with interlamellar layers, which are dependent on hydration and dehydration processes associated with material processing (Valkov and Simha 2012). *Trichoderma* can be easily mixed with vermiculite, by adding the concentrated spore suspension in water to the vermiculite matrix and mixing until the spores have the desired concentration of colony-forming units (CFU) per gram of vermiculite. Lewis et al. (1991) developed a formulation by mixing Terralite® grade 4 (a commercially available vermiculite) with wheat bran and conidia from several *Trichoderma* isolates. In the study, a method to activate the growth of the conidia was described, which includes moisture increments and acidification steps. The effects of activated/non-activated formulation, storage time (1–24 weeks), and temperature of storage (5 or 25 °C) were analyzed with respect to overall survival and saprophytic growth of *Rhizoctonia solani*. The study shows that when the formulation was added to the soil at a rate of 5% (w/w), there was a significant reduction in the survival of *R. solani*, which was enhanced when the activated formulation was stored at 5 °C. In another study by Martinez-Medina et al. (2009), *T. atroviride* C52 remained stable in a bentonite-vermiculite formulation for 8 weeks and significantly helped to reduce disease incidence (> 50%) of *Fusarium oxysporum* in *Cucumis melo* L. plants. Furthermore, in comparison to untreated controls, the treated plants showed an enhanced chlorophyll content and fresh/dry weights. Overall, vermiculite-based formulations are inexpensive, inert, and enhance moisture levels in soils, making them commercially interesting for *Trichoderma* spp. formulations either as a main carrier or as an adjuvant.

### Biochar-based formulations

Biochar is a light-weight and highly porous substance produced by biomass pyrolysis. It is made up of ash and carbon, but the exact final composition is defined by the type of biomass chosen, and the characteristics depend as well on the feedstock and on the production processes (Lee et al. 2018). Corn, rice, fruit peels, and wood from agricultural waste, as well as sludge and microalgae, are common feedstocks to produce biochar (Zhao et al. 2019). Several studies have shown positive effects of using biochar in biocontrol formulations, such as improving the soil conditions for nutrients and hence plant growth promotion (Biederman and Stanley Harpole 2013), increasing the number of viable *Trichoderma* CFUs, and reducing the incidence of diseases caused by phytopathogenic fungi (Muter et al. 2017; Akanmu et al. 2020)*.* Graber et al. (2014) summarized the available literature on biochar-mediated activities and highlighted possible mechanisms as to why the use of biochar may help to suppress the disease incidence of pathogenic fungi in plants. Likely, a combination of the alkalization of soil pH, improvement of water retention in soils and nutrient content, improvement of soil structure, and of soil microbiome all contribute to disease suppression. Some studies have also shown a synergistic effect of biochar and *Trichoderma* species that results in plant growth promotion (de Araujo et al. 2019b; Sani et al. 2020). In our own studies, the antagonistic potential of *Trichoderma harzianum* (strain T-720) was confirmed among other four *Trichoderma* spp. against five brown-rot basidiomycetes in dual culture tests. For this purpose, T-720 was genetically transformed and tagged with green fluorescent protein (Fig. 2E). We demonstrated that biochar amended with T-720 in a concentration of 10^5^ CFU g^−1^ dry biomass prevented weight losses in wood stakes by brown-rot fungi by almost 100% after 9 months of incubation (Ribera et al. 2017b). It was also demonstrated that T-720 inhibits the oxalic acid production by basidiomycetes, a well-known mechanism used by brown-rot fungi to detoxify Cu from impregnated wood (Fig. 2F).

However, biochar feedstock, particle size, and concentrations added to soil must be studied carefully, since some research has suggested that high concentrations can dramatically change the soil composition and lead to the promotion of pathogen growth. For instance, de Araujo et al. (de Araujo et al. 2019a) demonstrated that the application of biochar obtained from sewage sludge could reduce in vitro infections by *Macrophomina phaseolina* at concentrations of 1%, while higher concentrations of biochar potentiated *M. phaseolina* pathogenicity. They suggested that when biochar exceeds 1%, perturbations in the C/N ratios occur and may boost the growth of *M. phaseolina*. Regarding the effects of the particle size and feedstock of biochar, Vecstaudza et al. (2018) studied the influence of small (< 2 mm) and large (20–2 mm) particle-sized wood-derived biochar to the microbial community variations and in the soil composition. The study showed that independently of the particle size and the presence or absence of *Trichoderma viride*, the wood-derived biochar showed a significant increase in Ca^+2^ and Mg^+2^ concentrations, and depleted Al^+3^. Furthermore, they stated that the small (< 2 mm) particle-sized biochar was able to stimulate plant growth. Regarding *T. viride* viability, the survival increased with both sizes of biochar in the tested conditions, but especially when the small particle-sized biochar was used. Biochar can be a great option as dry carrier substance because it is easy to handle, and its application in fields not only helps to boost the sporulation of *Trichoderma* spp., but also improves the water-holding capacity and hence reduces fertilizer drainage in soils and promotes plant growth (Ulyett et al. 2014; Li et al. 2021; Wong et al. 2022).

## Liquid and gel formulations

**Fig. 3:**
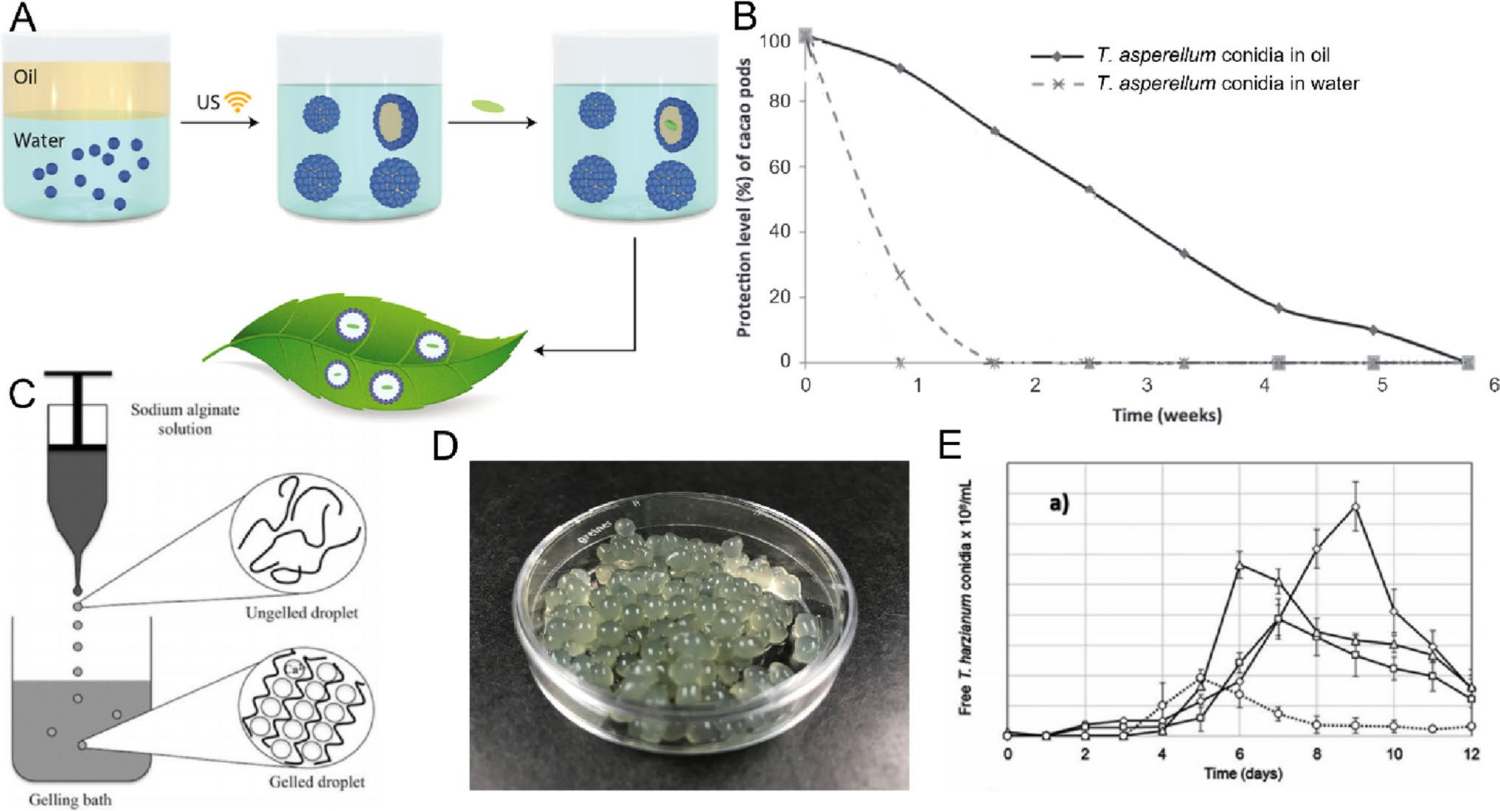
A summary of common liquid and gel-based formulations. **A** Schematic illustration of oil emulsion formulation preparation for spore encapsulation and application onto a leaf surface; reprinted with permission (Yaakov et al. 2018), copyright American Chemical Society 2018. **B** Protection efficacy of *Trichoderma asperellum* conidia when prepared in an oil-based formulation versus when prepared directly in water; adapted with permission (Mbarga et al. 2014), copyright Elsevier 2014. **C** Schematic illustration of alginate bead formation via extrusion into calcium solution; reprinted with permission (Ching et al. 2017), copyright Taylor & Francis 2017, and **D** photograph of *T. atrobruneum* T-720 spores encapsulated in 2% sodium alginate beads. **E** Growth of conidia when immobilized in alginate beads of various sizes (solid black lines) versus free conidia prepared directly in water (dotted grey line); reprinted with permission (Mancera-López et al. 2019), copyright Taylor & Francis 2019

Typically, liquid and gel-based formulations use an oil or water-soluble polymer network to stabilize encapsulated *Trichoderma* propagules in a relatively hydrated state. In comparison to dry formulations, the higher water activity in liquid and gel-based formulations makes it much more challenging to extend product shelf life due to spontaneous germination (Gervais et al. 1988), or to the aforementioned imbibition damage over time associated with prolonged exposure to water. Furthermore, in the liquid/gel state, products are more susceptible to bacterial contamination and therefore require a greater emphasis on sterile processing. For these reasons, current commercialized products are typically dominated by dry formulations. However, despite these shortcomings, with improving good manufacturing practices, and simple application of such formulations, liquid and gel-based formulations are rapidly gaining attention for industrial use. Here, current research has typically focused on techniques and modification processes for developing formulations that help maintain propagule viability for elongated storage periods (ca. > 6–12 months at ambient conditions). Common liquid and gel formulations (Fig. 3) including those based on oil encapsulation and encapsulation in natural polymer networks such as alginate are discussed below.

### Oil-based formulations

Oil-based formulations consist of a mixture of biocontrol propagules in oil, with or without the combination of water. The oil used can be mineral-based (extracted from crude oil) or vegetable-based (extracted from plant seeds) (Peng and Xia 2011). These formulations can be classified as oil emulsions (water-in-oil or oil-in-water), and oil dispersions. Oil emulsions consist of a mixture of two immiscible liquids; typical emulsions for biocontrol products are oil-in-water, whereby the biocontrol propagules (e.g., conidia) are contained in the oil phase. Here, emulsifiers are often needed, which help in dispersing the propagule-oil mixture once the dilution in water takes place before application (Xavier-Santos et al. 2011). Conversely, oil dispersions consist of a non-aqueous suspension of the biocontrol propagules inside the mineral or vegetable oil (Swarnakumari et al. 2020). In general, oil-based formulations are quickly gaining attention on the biocontrol market, which is likely due to their simple use, better resistance to leaching by water (Luz and Batagin 2005), improved UV tolerance (Fernandes et al. 2015), and protection against incompatible water-soluble chemical pesticides (Lopes et al. 2011). Vegetable oil and mineral oil-based formulations have proven to be good formulations for biocontrol activities of enthomopathogenic fungi against target arthropods (Perinotto et al. 2017) and have superior thermal stability when compared to conidia in water suspensions (Paixão et al. 2017).

**Fig. 4:**
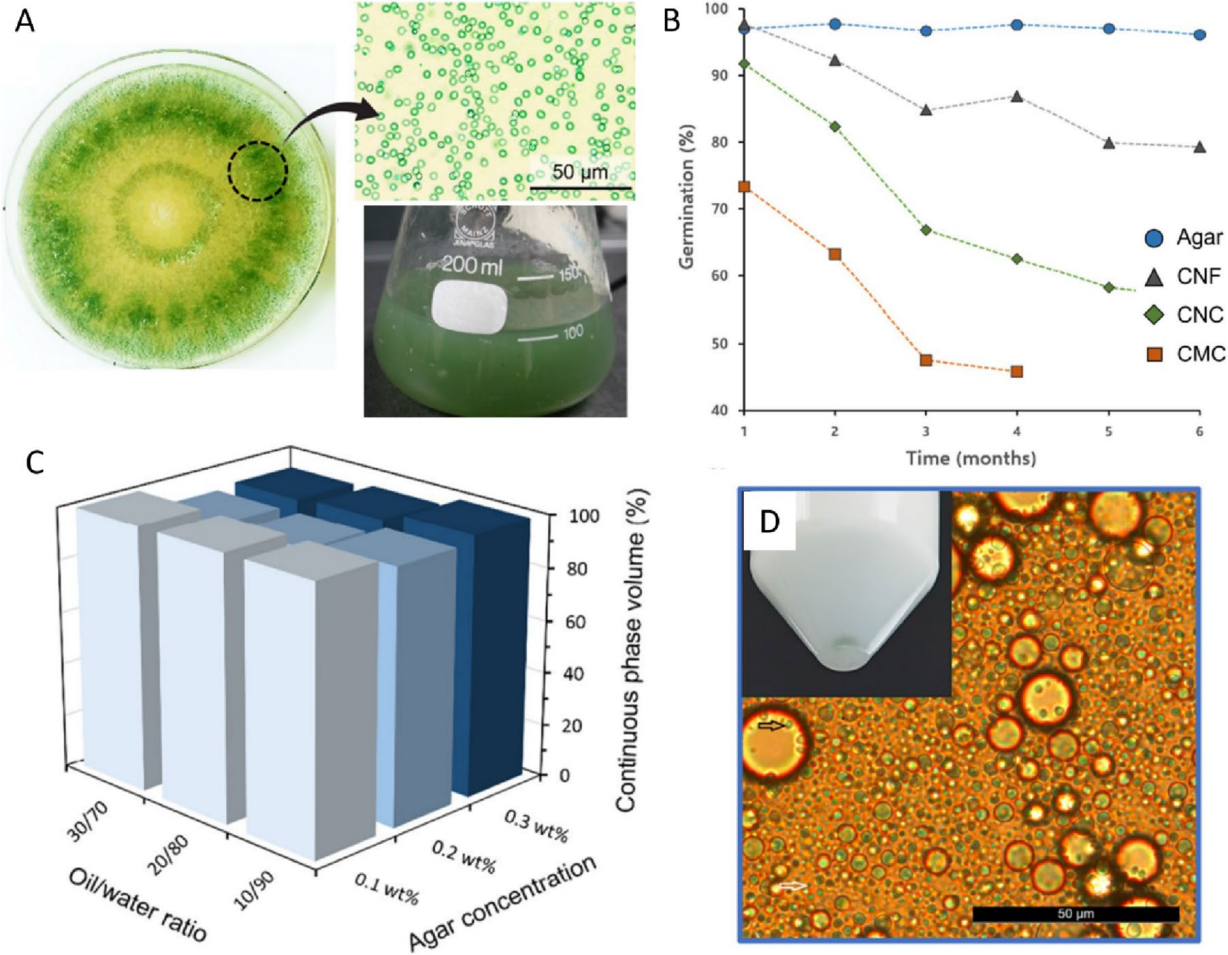
Preparation of biopolymer-stabilized emulsions for encapsulating *T. atrobrunneum* 720 conidia. **A** Schematic illustration of the preparation of conidia suspensions. **B** Germination of conidia over time in various biopolymer solutions. **C** Canola oil-in-water emulsion stabilization in agar-based formulations, as determined via measuring the continuous phase volume percent after 28 days. The effects of oil:water ratio and agar concentration are shown. **D** Photograph and microscopy image of *T. atrobruneum* T-720 spores encapsulated in 0.1% agar-stabilized canola oil-in-water emulsions with a 30:70 oil:water ratio; adapted with permission (Martínez et al. 2023), copyright Springer Nature 2023

Regarding *Trichoderma* spp. formulations, Mbarga et al. (2014) showed that *T. asperellum PR11* conidia in an oil dispersion formulation composed mainly of soybean oil showed 50% conidial germination after 22.5 weeks, stored at 25 °C. Interestingly, when applied in the field, the formulation was able to protect cacao pods against *Phytophthora megakarya* more effectively than some common synthetic fungicides. In another study by Akshata et al. (2018), a formulation based on paraffin oil was tested for *T. viride* conidia. The survival rates after 6 months of storage at 27 °C decreased from 2.8 × 10^9^ CFU mL^−1^ to 1.8 × 10^9^ CFU mL^−1^, and growth inhibition was approximately 80% for *Fusarium oxysporum f.* sp. *Ciceri*, and *Sclerotium rolfsii*, and 85% for *Rhizoctonia bataticola*. Herrera et al. (2020) developed emulsions consisting of commercial vegetal oils (Vatel® and Surfatron®) and mineral oils (Aceite Blanco®) with *T. asperellum* TV190 conidia. The results indicate that these emulsions can improve the antagonistic potential against *Rhizoctonia solani* under greenhouse conditions when compared to treatments of conidia in water. Furthermore, the viability of conidia was higher (56–63% for vegetable oils) when compared to conidia in water (8–12%) after exposure to UV irradiation. Recent work in our group investigated the use of various biopolymers to stabilize *T. atrobrunneumm* 720 conidia within the oil phase of oil-in-water emulsions (Fig. 4). Here, both agar and cellulose-nanocrystal-based formulations demonstrated ca. 100% viability of encapsulated conidia after 1 month of storage at ambient conditions, dropping to 70% and 40% respectively after 6 months of storage (Martínez et al. 2023). The effects of biopolymer concentration, oil: water ratio, and oil type were all investigated; however, the biopolymer itself demonstrated the greatest effect on conidia viability and emulsion stability. In summary, oil-based formulations show high viability levels at extended storage times, have a protectant effect against UV radiation, improve biocontrol performance, and can help to improve adhesion to hydrophobic surfaces. The latter is very important for foliar application of the products (Birnbaum et al. 2021). Due to these positive aspects, oil formulations have great potential as carrier substances for developing high-quality biocontrol products, although increased cost and production complexities hinder their current practical commercial use.

### Alginate-based formulations

Alginate is a non-toxic biodegradable hydrocolloid that can form thermally stable hydrogel beads in the presence of divalent cations, such as calcium. They are commonly used to encapsulate microbial cells, enzymes, hormones, drugs, oils, herbal extracts, and flavors (Szekalska et al. 2016). It is important to mention that the success for manufacturing alginate beads depends on size variation, shape, biocompatibility, swelling, solubility, mechanical, and chemical stability (Lee et al. 2013). Subject to the final size of the droplets, they can be differentiated as follows: macrogels > 1 mm (normally produced by extrusion), microgels (0.2 to 1000 µm) and nanogels (< 0.1 µm). Microgel particles are the most used for biocontrol applications, and several methods have been developed for their production: modified extrusion dripping, microfluidics, emulsification, impinging aerosol, and electrostatic atomization (Ching et al. 2017). Several studies have investigated the potential of *Trichoderma* conidia encapsulated in alginate (Fig. 3D). For instance, Mancera-Lopez et al. (2019) successfully encapsulated *T. harzianum* propagules in different-sized calcium alginate beads by the dripping method (average diameters of 1.5 ± 0.3 mm, and 2.7 ± 0.3 mm), and by internal gelation (diameter 8.6 ± 3 µm), using them to produce conidia in submerged fermentation using a bioreactor. Beads were dried at 40 °C and stored at room temperature, showing a minimum viability value of 70% after 2 years of storage. Furthermore, the capsules of 1.5 ± 0.3 mm diameter led to the highest count of free conidia (1.5 × 10^8^ ± 0.2 × 10^8^ conidia/mL) after 9 days of incubation. This concentration was 36% and 87% superior to those in the capsules of 8.6 ± 3 µm and 2.7 ± 0.3 mm diameter, respectively. These results are in good agreement with other studies, which demonstrate the positive effect of Ca^2+^ ions in the sporulation rates (Šimkovič et al. 2008; Jurić et al. 2019). Other positive aspect on the encapsulation in alginate is that enzymatic activities involved in biocontrol mechanisms are not negatively affected. For example, Maruyama et al. (2020) encapsulated *T. harzianum* in alginate beads and showed that, besides increasing photostability against UV radiation, chitinase and cellulase activities were maintained or even increased after encapsulation, and the encapsulated propagules showed a high antagonistic potential in vitro against *Sclerotinia sclerotiorum.* In another study, Locatelli et al. (2018) analyzed the impact of different blends of sodium alginate and other polymers and studied their interactions during the ionic gelation process to encapsulate *Trichoderma* conidia. They demonstrated interactions between the alginate matrix and different polymers used for formulations, with the blend of alginate and starch exhibiting the highest conidial viability during drying. After 14 months of storage at 28 ± 2 °C, the mixture that showed the highest spore viability (2.4 × 10^6^ CFU/g) was 2% sodium polyphosphate, 2% citrus pectin, and 2% glycerol. Alginate capsules can be disrupted to release the encapsulated conidia by adding them into a solution of 2% sodium citrate. These studies show that *Trichoderma* spores can yield outstanding viability levels over storage time when they are encapsulated in alginate beads. Furthermore, the beads are inexpensive to produce, biodegradable and non-toxic, and have a positive impact on biocontrol enzymatic activities, protection against UV light, and sporulation rates, being thus an excellent option to develop biocontrol formulations.

## Drying techniques

Dry formulations are the most commonly used for encapsulating *Trichoderma* and other BCAs, dominating the market for registered products. The main reason for this is the inherent stability of the products encapsulated within dry formulations; in comparison, liquid formulations change properties (density, pH, concentration, etc.) over time due to evaporation and other solvent effects, which makes product registration complicated. Some of the other advantages of dry formulations are a lower susceptibility to contamination compared to liquid formulations, and the preservation of the spores in a status that mimics the original environment in nature, avoiding imbibition damage. All drying techniques have the goal to reduce moisture content while providing coated propagules/particles. The dry light-weight formulations reduce transportation costs, prolong shelf life, and avoid the need for low storage temperatures (Jones and Burges 1998). The most studied drying techniques are freeze drying (lyophilization), fluidized bed drying, and spray drying (Table 3). For these techniques, the coating material composition and rehydration processes are critical to reach high survival rates of the microorganisms, as both factors are involved in cell protection and physiological recovery (Carbó et al. 2017). Depending on the drying procedure, cryoprotectants or osmoprotectants might be required; several components have been successful for the cryopreservation of microorganisms, such as dimethylsulfoxide, glycerol, serum albumin, skimmed milk, peptone, and sucrose (Hubálek 2003). Jin et al. (2009) demonstrated that the hydrophobicity of conidia plays an important role by improving resistance to the drying procedure and improving rehydration behavior. Furthermore, response surface methodologies applied to drying techniques for biocontrol products can be helpful in order to adjust the parameters that have the greatest influence on fungal viability (Aguirre-Güitrón et al. 2018). In the following sections, we describe different drying techniques, with an emphasis on their benefits for developing biocontrol products with *Trichoderma* propagules.

**Table 3:** Summary of the drying techniques used for the development of *Trichoderma* spp. formulations. In each case, processing parameters, type of propagules, additives and spore viability are listed

Drying technique	Processing parameters	Conidia species	Additives	Spore viability (%)	Ref
Spray drying	Inlet/outlet temp 90/65 °C	*T. asperellum*	Maltodextrin DE20	93% after drying	(Braga et al. 2019)
Spray drying	Inlet/outlet temp. 80/50 °C	*T. harzianum*	-	25% after drying	(Fernández-Sandoval et al. 2012)
Spray drying	Inlet/outlet temp. 60/31 °C	*T. harzianum*	Sucrose	70% after drying	(Jin and Custis 2011)
Spray drying	Inlet/outlet temp. 150/90 °C	*T. harzianum*	Maltodextrin DE10	86% after drying	(Muñoz-Celaya et al. 2012)
Fluidized bed drying	*50 °C until a moisture of 10%*	*T. atroviride*	Lignocellulose carrier	Up to 100% after drying	(Witkowska et al. 2016)
Freeze drying	Frozen at − 26 °C, then dried at pressure of 0.2 mbar for 20 h	*T. atroviride* *T. harzianum*	-	Up to 100%	(Grzegorczyk et al. 2018)
Freeze drying	frozen at − 80 °C over night, then vacuumed at 300 mT for 48 h	*T. asperellum*	Calcium alginate	From 6.6 × 10^7^ conidia g^−1^ to 1.1 × 10^7^ conidia g^−1^ after 120 days storage at 8 °C	(Lopes et al. 2020)

### Freeze drying (lyophilization)

Freeze drying or lyophilization is a 2-phase process, in which a material is initially frozen, and subsequently, the solvent present (normally water) is removed by sublimation under vacuum, ideally to a final level low enough to avoid biological growth or chemical reactions (typically between 1 and 6% moisture content) (Morgan and Vesey 2009). This technique has been used successfully for decades as a preservation method for fungal cells (Davies 1962). The major shortcomings are the significant loses in conidial viability during processing, frequent cross-contaminations, and relative lack of scalability of the freeze-drying process (Barbaree and Sanchez 1982). Nevertheless, this process has been used to great effect, with viability losses mostly dependent on the type and morphology of the cell structures under study (Tan et al. 1994). Berny and Hennebert (1991) studied the influence of freezing on the viability of yeast cells and conidia from different filamentous fungi. *T. viride* conidia showed the best viability results, which were never less than 85%, regardless of the freezing rate (from 1.6 to 40 °C min^−1^) and the cryoprotectants used. Some studies have indicated that the moisture content must be reduced to a minimum to avoid activation of resting dry conidia, thereby retaining viability (Michaelsen et al. 2013). A combination of media components, freezing rates, as well as dehydration and rehydration, and the addition of lyoprotectants and cryoprotectants seems to have a significant impact on conidial viability (Croan 2000). For example, trehalose, maltodextrin, and skimmed milk are good for stabilizing cell membranes during dehydration by acting as bulking agents substituting polar boundaries of water, and protecting against protein denaturation (Crowe et al. 1987; Tan et al. 2007). Grzegorczyk et al. (2018)studied the effects of lyophilization and the addition of maltodextrin on four strains of *T. atroviride* (TRS14, TRS25, TRS40, TRS43), a strain of *T. harzianum* (TRS85) and two strains of *T. virens* (TRS106 and TRS109). The samples were first frozen at − 26 °C, then dried under pressure at 0.2 mbar for 20 h, and finally stored at room temperature. After 3 months of storage, enzymatic activities were very different when compared with the initial values. Although xylanolytic enzymes increased significantly after lyophilization, the production of cellulolytic enzymes did not increase significantly for most of the strains, and the pectinolytic activities were lower for all strains tested. Interestingly, maltodextrin affected the viability depending on the species and strains: for two of the *T. atroviride* strains (TRS14, and TRS40), there were significant differences in conidial viability when maltodextrin was used. After 3 months of storage, the concentration of the strain TRS14 prepared in only water was 3.11 × 10^7^ CFU g^−1^ and was 8.69 × 10^7^ CFU g^−1^ when maltodextrin was added. For the strain TRS40, the number of viable spores was higher, yet lower than for the strain TRS14 (4.39 × 10^6^ for the control 1.31 × 10^7^ for the treatment with maltodextrin). There were no significant differences for the rest of the tested strains in comparison to the controls. It is worth mentioning that the viability varied dramatically with species and among strains under the same conditions studied, without showing a negative effect on viability by maltodextrin. In a novel approach, Lopes et al. (2020) compared the stability of conidia of unfrozen and frozen alginate beads containing *T. asperellum* BRM-29104. The conidia were frozen at − 80 °C overnight and then transferred to a lyophilizer with a vacuum pressure of 300 mT for 48 h at − 50 °C. After 120 days of storage at 8 °C, viable conidia in the unfrozen beads decreased from 1.2 × 10^8^ to 1.4 × 10^7^ conidia g^−1^, while the viability loss was lower for the frozen samples, decreasing from 6.6 × 10^7^ conidia g^−1^ to 1.1 × 10^7^ conidia g^−1^. Overall, freeze drying is a simple but well-studied preservation method to develop dry formulations and products for biological control. Moving forward, deeper research on carrier materials should be considered to further improve this method of encapsulation for *Trichoderma* spores.

### Fluidized bed drying

Fluidized bed drying functions by spraying air upwards in an enclosed in a container, while the product flows along a belt/bed, resulting in a granulated product (Morgan et al. 2006). In general, there are two main phases during the drying process: initially drying is continuous and independent of the moisture content, and in the later phase the moisture content is reduced (Amador and Martin de Juan 2016). The kinetics of drying is essential in order to estimate the time required to reduce moisture content appropriately (Srinivasakannan and Balasubramanian 2002) and can be modified by the atomizing air pressure, the spray time and rate, and the bed temperature (Stummer et al. 2012). The use of adjuvents is also important here to ensure the high viability of the BCA propagules during exposure to elevated temperatures associated with the drying process. Several studies have monitored these crucial parameters and performed mathematical modelling to optimize the drying of fungal products (Arumuganathan et al. 2009; Darvishi et al. 2019). In studies by Bayrock and Ingledew (1997), a mathematical approach was used to determine the viability of the yeast *Saccharomyces cerevisiae*. The study demonstrated that the dehydration procedure was the greatest factor that negatively influenced viability, but not the heating or oxidation processes. Moreover, they concluded that the viability of cells dropped rapidly when the moisture content was less than 15% during the falling-rate period. Some dehydration protectants mentioned above for freeze drying, such as trehalose, proved to minimize spoilage during dehydration by fluidized bed drying. In an investigation by Larena et al. (2003), conidia of *Epicoccum nigrum* was dried using a range of temperatures between 30 and 40 °C. The conidial viability was maintained above 80% after 3 months storage at room temperature and then started to decrease to 75% and 37% after 120 and 150 days of storage, respectively. Witkowska et al. (2016) tested the viability of dried *T. atroviride* TRS40 conidia after 3, 6, and 12 months of storage at room temperature. For this experiment, *T. atroviride* TRS40 was cultivated on two lignocellulose substrates, and the resulting conidia were harvested and preserved by fluidized bed drying at three different inlet temperatures: 50, 60, and 70 °C, for 1 to 2.5 h until reaching a moisture content below 10%. During the drying process, the viability rate was maintained at 100% only with the lowest temperature (50 °C). At the highest temperature (70 °C), viability was reduced to 40.4% and 76.3% (depending on the substrate). However, this method resulted in a high loss of conidial viability over time. After 3 months of storage, the viability was reduced at a rate of 14.5–38.0%. No significant reduction in viability was recorded after 3 to 6 months of storage, whereas after 6–9 months, a reduction in viability by 4.1–19.2% was recorded. Although fluidized bed drying can be performed continuously (unlike freeze drying), the drying times required for fluidized bed drying are much longer than needed for other drying techniques. This feature has a negative impact on production cost from an industrial perspective. However, with proper product encapsulation, fluidized bed drying is a relatively mild processing technique, demonstrating relatively high BCA viability post-processing. Moving forward, systematic optimization of processing temperatures and times could make this technique an interesting choice to formulate *Trichoderma* products for practical biocontrol applications.

### Spray drying

**Fig. 5:**
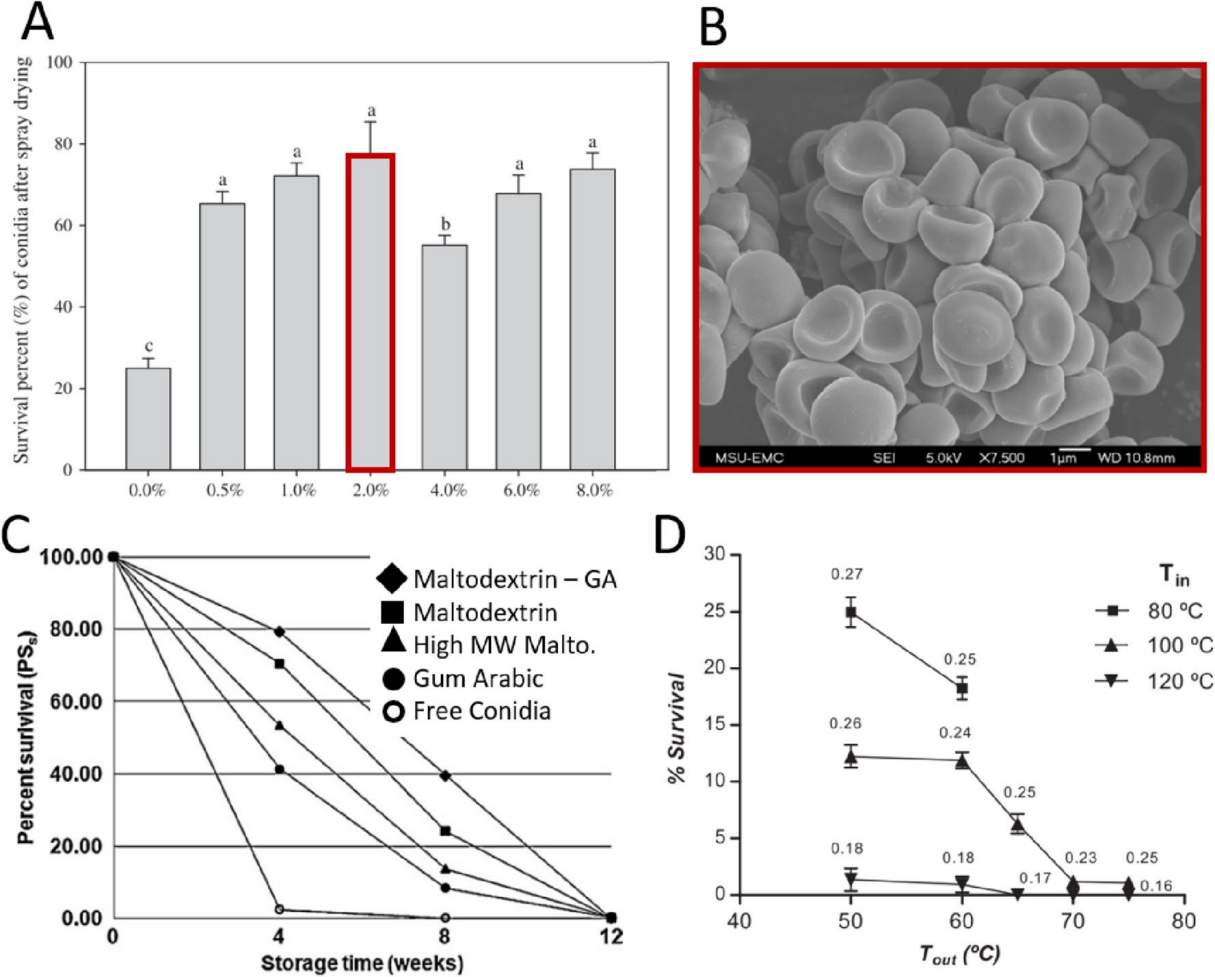
Effects of spray drying on *Trichoderma harzianum* conidia. **A** Initial survival of conidia spray dried using varying concentrations of sucrose as an excipient, and **B** morphology of resulting spray dried particles via SEM; adapted with permission (Jin and Custis 2011) copyright Elsevier 2011. **C** Effects of polymer excipient on conidia survival over time following spray drying; adapted with permission (Muñoz-Celaya et al. 2012), copyright Elsevier 2012. **D** Effects of inlet and outlet temperature on initial conidia survival; adapted with permission (Fernández-Sandoval et al. 2012) copyright Elsevier 2012

Spray drying is a technique whereby a suspension formulation is atomized and sprayed under pressure into a heated chamber, forming dried particles, which can then be collected (Show et al. 2019). In contrast to freeze drying and fluidized bed drying, the thermal shock is extremely short due to the rapid processing speed. This enables much higher temperatures to be used, which may otherwise drastically reduce the viability of BCAs. In a comprehensive review, Santos et al. (Santos et al. 2018b) described a number of parameters such as inlet–outlet temperatures, gas flowrate, feedstock flow rate, viscosity, and atomization pressure, which can all adversely influence the quality and features of a given product processed via spray drying. Albeit, spray drying is a quick, highly automatized, and scalable processing technique which has already shown much industrial success for the encapsulation of other temperature-sensitive materials such as polymer nanoparticles for drug delivery (Saini et al. 2018), food powders (Woo and Bhandari 2013), and various microorganism for different purposes (Schuck et al. 2013). Therefore, we anticipate that spray drying could also have a great potential to produce light-weight *Trichoderma* biocontrol products (Fig. 5).

However, there are some drawbacks for this method of microbial encapsulation, which include the loss of conidial viability due to elevated processing temperatures and shear during atomization, and the production of dust, both of which should be carefully taken into account during large-scale production and packaging processes. Importantly, the sensitivity of propagules during drying/heating and the selection of spray drying parameters will have an impact on propagule viability. Fernández-Sandoval et al. (2012) determined that among all parameters involved during the spray drying process, thermal stress was the main factor that affected survival of *T. harzianum* conidia. Using outlet/inlet temperatures of 50/80 °C respectively, the viability of conidia was reduced significantly (> 75%) when compared to the viability of controls. The optimization of the inlet/outlet temperatures and the use of carrier substances that protect the biological material from heat stress could counteract the viability loses. As an example, Jin and Custis (2011) determined that by adding 2% (w/v) sucrose to base suspensions, a survival rate for *T. harzianum* conidia of 70% was obtained, representing an increase of almost threefold. In another study by Pérez-Alonso et al. (2003), different maltodextrin polymers, both as single emulsions and as mixtures, were investigated for their effect on conidia protection against heat damage. They demonstrated that there was a statistical correlation between the activation energy (Ea) of the carbohydrate polymers and lower heat damage to *T. harzianum* conidia. They suggested that studying Ea of different coating materials could serve as a criteria for selecting the best biopolymer matrix during spray drying. Muñoz-Celaya et al. (2012) suggested that the dryer’s feed flow could be increased at the highest temperatures, and thus the conidia would be exposed for a shorter time to heat and thermal stress. In their experiments, high inlet/outlet temperatures provided the best survival rate for *T. harzianum* conidia after spray drying (up to 86% using 150/90 °C) when they used maltodextrin and gum arabic as a matrix. This demonstrates the importance of dryer feed flow right on conidial survival and proves that lower temperatures are not necessary to ensure high survival. The authors proposed that higher inlet/outlet temperatures are necessary in order to generate a consolidated matrix of coating material. However, other authors indicate that a lower range of temperatures can be applied as well for microencapsulating fungal conidia in dextrin and other polysaccharides, obtaining good viability results (Liu and Liu 2009). Their investigations stated that applying 60/30 °C for the inlet/outlet temperatures, and a matrix of dextrin (10% w/v), 10% skimmed milk, and 5% PVP K90 as carrier substances, conidial viability of *Beauveria bassiana* was up to 80% after 6 months storage at 4 °C. In a more recent study, Braga et al. (2019) spray dried conidia of *T. asperellum* using different coating materials in order to determine appropriate processing temperatures. Here, inlet temperatures > 90 °C had the highest lethal effect on conidia (12.89 ± 2.37% survival rate at 100 °C if no coating material was used). At processing temperatures of 90/65 °C, the highest survival rate of conidia (92.89 ± 1.47%) was obtained using maltodextrin DE20, followed by whey powder (82.84 ± 2.35%). Although spray drying is a relatively quick and scalable technique for the development of dry biocontrol products, the relatively low amount of viable spores after the drying process (as compared to other drying techniques) is a major drawback associated with this method. However, as with fluidized bed drying, systematic optimization of processing temperatures and times could lead to improved viability rates, and thus increased use of spray drying processes in commercial formulations.

## Summary and future outlook on biotechnological approaches to improve *Trichoderma*-based products

The development of new *Trichoderma-*based products shows great promise in the context of sustainable agriculture and arboriculture practices. In this review, we have underlined the current insights in product formulations and the potential of several carrier substances and processing techniques to innovate and improve biocontrol performance and product properties. For instance, investigations on drying and microencapsulation techniques, which aim to increase product shelf life and stability, will enable the production of superior biocontrol products and pathogenic control. Future work in this area should focus on the progressive adaptability of these processes for the encapsulation of conidia by testing new coating substances that protect the spores against high temperatures, various drying methods, and during the rehydration/application processes. Of equal importance are studies on other inexpensive biodegradable materials, targeted by enzymes that are highly expressed by a wide range of phytopathogenic fungi. In this regard, some of our own studies investigated the differences in 6 different biopolymer carriers for *T. atrobrunneum* conidia, showing the use of agar at low concentrations (0.2%) leads to exceptional stability (96% germination rates for conidia after 6 months of storage at 22 ± 2 °C) (Martínez et al. 2023). We attributed this pronounced improvement in the viability of agar-based formulations as compared to cellulose-based formulations to the expression of cellulolytic enzymes in *Trichoderma* (Zhou et al. 2008; Shafique et al. 2009; Colussi et al. 2011). We hypothesize that cellulose and its derivatives could trigger the secretion of degradative enzymes and the spontaneous germination of the spores. On the other hand, although agarases are expressed by some bacteria (Fu and Kim 2010; Wenjun et al. 2016; Khalifa and Aldayel 2019; Fawzy et al. 2020), they are not known to be expressed by *Trichoderma*, likely contributing to spore dormancy. Nevertheless, formulations containing low concentrations of cellulose nanocrystals and other biopolymers, such as lignin, pectin, or cutin could promote the expression of important cell wall-degrading enzymes that are involved in biocontrol activities by *Trichoderma* spp. in field (Geraldine et al. 2013; Giovannoni et al. 2020). As a result, these carrier substances could contribute to improved efficacy and activity in biological control, an important feature when the target pathogen is very aggressive.

Furthermore, alternative encapsulation and surface coating techniques could be further investigated to improve the stability of *Trichoderma* biocontrol products, such as Pickering emulsions and layer-by-layer (LbL) assembly. Pickering emulsions rely on solid particles to stabilize droplets (Yang et al. 2017), and to date have been popular in the development of formulations for drug delivery (Tai et al. 2020), food applications (Berton-Carabin and Schroën 2015), cosmetics (Wei et al. 2020), pharmaceutical applications (Albert et al. 2019), and to encapsulate microbial cells (Van Wijk et al. 2014). Besides our recent study using cellulose nanocrystals, (Martínez et al. 2023) there are currently no examples of Pickering emulsions for the encapsulation of *Trichoderma* products. However, some studies have investigated their use to encapsulate other microorganisms for biocontrol applications. Bashir et al. (2016) encapsulated *Bacillus thuringiensis*, which is used as entomopathogenic organism, by water-in-oil Pickering emulsions. They designed colloidosomal and pH-sensitive microparticles (50 µm) that could release the *B. thuringiensis* in the mid gut of lepidopteran larvae (for pH > 8.3). The microencapsulated bacterial cells more effectively decreased the number of larvae after 12 days compared to the chemical pesticide λ-cyhalothrin. Yaakov et al. (2018) tested oil-in-water Pickering emulsions to encapsulate conidia of the entomophatogenic fungi *Metarhizium brunneum* using functionalized silica nanoparticles and paraffin oil. Compared to controls of conidia in 0.01% Triton and in distilled water, the encapsulated conidia had a better coverage when sprayed on the surface of *Ricinus communis* leaves, and higher mortality rate in *Spodoptera littoralis* larvae. Promisingly, the germination rate of the encapsulated conidia was 85 ± 8.3% after emulsification, as compared to 95 ± 5% for the non-encapsulated conidia. Albeit, the conidia remained alive in the oil droplets for only 3 weeks, which underlines the need to improve the shelf life of such novel formulations. Given the successes of Pickering emulsification in other applications, and the ability to incorporate additional functionality (such as stimuli responsiveness or adhesiveness), this technique represents a promising and relatively straight-forward method of encapsulating viable microorganisms for next-gen biocontrol.

The layer-by-layer (LbL) technique is based on the consecutive assembly of nano/micro-sized layers of material (e.g., polyelectrolites, nanoparticles, proteins) to form stable core–shell type particles (Hua and Lvov 2007). Adhesion between the assembled layers may be based on ionic, electrostatic, hydrogen bonding, coordination, or hydrophobic interactions, contributing to the versatility of this technique (Zhang et al. 2007). Some of the most relevant fields of application of LbL-based materials are as responsive drug delivery systems (Johnston et al. 2006; Santos et al. 2018a), cell surface engineering (Custódio and Mano 2016), and biofunctionalization of inert surfaces (Hartmann and Krastev 2017). Furthermore, it has been successfully used to encapsulate microorganisms (Franz et al. 2010; Fakhrullin and Lvov 2012; Lee et al. 2015; Anselmo et al. 2016) for different purposes. For example, Fakhrullin et al. (2009) coated *Saccharomyces cerevisiae* cells and *T. asperellum* conidia with polyelectrolytes and gold and silver metal nanoparticles to confer beneficial optical, electrical, and magnetic properties. They also demonstrated that both the cells and the conidia were alive after the coating process, and that the polyelectrolyte layers protected them against toxicity of the silver and gold nanoparticles. In a recent paper by Peil et al. (2020), *T. reesei* was encapsulated following the layer-by-layer assembly using kraft lignin and modified cationic kraft lignin to form a polyelectrolyte capsule. The lignin shell was non-toxic for the spores, as they were able to maintain germination rates comparable to the non-encapsulated controls. Furthermore, this design allowed the germination of the coated spores in a responsive manner to enzymatic activities of esca pathogens. The application of culture filtrates of *Phaeomoniella chlamydospora* and *Phaeoacremonium minimum* containing their secreted enzymes was able to break the lignin shell and trigger germination of *T. reesei* spores, at similar rates to the uncoated spores. However, LbL is a relatively time-consuming process, depending on the number of layers and the chosen coating technique, as it may involve several washing and centrifugation steps. Advanced encapsulation techniques such as Pickering emulsification and LbL that are able to preserve the stability of spores and fine-tune the properties of the applied products (i.e., adhesion, stimuli responsiveness) open new horizons for the development of formulations able to cover the major drawbacks in current biocontrol products. Overall, although research into the development of novel biological control strategies, formulations, and processing techniques is relatively well-established, new approaches in these areas are likely to lead to drastic improvements in performance and an increase in the commercialization and industrial use of *Trichoderma*-based products for biocontrol.
